# The use of soft silicone solid implant molded intraoperatively for *pectus excavatum* surgical repair

**DOI:** 10.1590/S1679-45082014AO2919

**Published:** 2014

**Authors:** Jaime Anger, Raphael Francisco Vesterman Alcalde, Jose Ribas Milanez de Campos

**Affiliations:** 1 Hospital Israelita Albert Einstein, São Paulo, SP, Brazil; 2 Hospital das Clínicas, Faculdade de Medicina, Universidade de São Paulo, São Paulo, SP, Brazil

**Keywords:** Funnel chest, Prosthesis and implants, Silicones, Chest wall/surgery, Thoracic surgical procedures/methods

## Abstract

**Objective:**

To describe a new surgical technique to treat *pectus excavatum* utilizing low hardness solid silicone block that can be carved during the intraoperative period promoting a better aesthetic result.

**Methods:**

Between May 1994 and February 2013, 34 male patients presenting *pectus excavatum* were submitted to surgical repair with the use of low hardness solid silicone block, 10 to 30 Shore A. A block-shaped parallelepiped was used with height and base size coinciding with those of the bone defect. The block was carved intraoperatively according to the shape of the dissected space. The patients were followed for a minimum of 120 days postoperatively. The results and the complications were recorded.

**Results:**

From the 34 patients operated on, 28 were primary surgeries and 6 were secondary treatment, using other surgical techniques, bone or implant procedures. Postoperative complications included two case of hematomas and eight of seromas. It was necessary to remove the implant in one patient due to pain, and review surgery was performed in another to check prothesis dimensions. Two patients were submitted to fat grafting to improve the chest wall contour. The result was considered satisfactory in 33 patients.

**Conclusion:**

The procedure proved to be fast and effective. The results of carved silicone block were more effective for allowing a more refined contour as compared to custom made implants.

## RESUMO

**Objetivo:**

Descrever a técnica para reparação de *pectus excavatum* com o uso de bloco de silicone sólido de baixa dureza, que possibilita a adequação de suas dimensões no intraoperatório para melhor resultado estético.

**Métodos:**

Entre maio de 1994 e fevereiro de 2013, pacientes do gênero masculino, portadores de *pectus excavatum*, foram submetidos à correção cirúrgica com bloco de silicone sólido de baixa dureza, de 10 a 30 Shore A, pré-fabricado em forma de paralelepípedo, com as dimensões da altura e da base coincidentes com as da falha óssea. Durante o ato cirúrgico, esse bloco foi esculpido até adequar-se ao formato do espaço dissecado. Os pacientes foram acompanhados por um mínimo de 120 dias de pós-operatório.

**Resultados:**

Trinta e quatro pacientes foram operados. Destes, 28 foram de tratamento primário e 6 secundário a outras técnicas cirúrgicas, ósseas ou de uso de implantes. As complicações foram dois casos de hematoma e oito de seroma. Foi necessária a retirada do implante em um dos casos devido à dor. Em outro caso, foi a realizada revisão cirúrgica das dimensões da prótese. Dois pacientes foram submetidos a enxerto de gordura, para melhorar o contorno da parede torácica. Não ocorreu nenhum caso de infecção. O resultado foi considerado satisfatório em 33 pacientes.

**Conclusão:**

O procedimento mostrou-se rápido e efetivo. Os resultados dos contornos obtidos foram considerados mais efetivos quando comparados aos obtidos com a utilização de próteses pré-moldadas.

## INTRODUCTION

Congenital deformities of the chest wall include several musculoskeletal defects that change the thoracic symmetric contour. *Pectus excavatum* is the name used to describe a depression in which the sternum grows inwards near the back cartilages of the spine. The most frequent malformation occurs in the medial region of the anterior chest and affects half or two thirds of the inferior portion of the sternum, with maximum recess at the junction of the chest with the abdomen. This abnormality is more common in males.^(1)^

The treatment may be surgical or, non-invasively, by using braces.^(1)^ Surgical options include mobilizing osteo-cartilaginous tissues with the purpose of placing them at the desired area, or those that consist of filling out the existing defect, usually using silicone.^(2–4)^

Today the most common surgical technique uses a metal bar and was described by Nuss.^(3)^ Silicone is used in selected cases, as when the deformity is considered mild, and when bone mobilization surgeries were unable to completely correct the contours.^(1)^

The many techniques using silicone implants are based on making a mold of the chest defect prior to surgery, which is then used to make the definitive implant. This implant can be solid or filled with gelatinous silicone.^(5)^

However, after using first solid silicone elastomer, and later gelatinous implants, we noticed that the results were not satisfactory. The pre-molded implant, based on the existing malformation, not always coincided with the dimensions of the actual defect, and that could only be seen intraoperatively, after dissecting the necessary site and placing the prosthesis. It was not possible to make any final changes on those implants.

In 1994 we started using soft solid silicone blocks that, after being sterilized, can be sculpted during surgery, enabling it to be molded according to the actual malformation found. In this article, we present our surgical experience in treating *pectus excavatum* in male patients.

## OBJECTIVE

To describe the surgical technique using soft silicone block sculpted intraoperatively to correct *pectus excavatum* in male patients.

## METHODS

From May 2nd 1994 to February 1st 2013, a retrospective study was carried out on male patients diagnosed with *pectus excavatum* with no other concomitant thoracic muscle abnormalities. These patients had been submitted to surgery using solid silicone after ruling out indication for bone repair or treatment with external compression braces. The study included patients with a minimum of 120 days postoperative follow-up.

Notes were taken on the complications and results. The aesthetic result was split into satisfactory or unsatisfactory, according to the patient's assessment. The submitted project was considered as case series by the Research Ethics Committee from the *Instituto Israelita de Ensino em Pesquisa*, which considered the use of this technique an individualization and improvement of an already standardized procedure, without causing risks or implications on prognosis or hospital length of stay. For that reason they decided a prior Informed Consent was not necessary.

### The implant

During the pre-operatory period, the bone defect dimensions were written down, and a soft solid silicone block was ordered, hardness between 10 and maximum 30 Shore A, shaped as a parallelepiped. The base measures are equivalent to the defect maximum measures and the block's height is the same as the defect maximum height.

### Surgical technique

General anesthesia was used with local infiltration, using 400 mg lidocaine solution at 0.5%, adding adrenaline at 1:200,000. All patients stayed at the hospital for a maximum of 12 hours after surgery.

The initial stage of surgery began with marking the defect edges on the chest, with the patient standing up or in supine position. Next, the incision site was marked. The incision could be on existing scars from secondary surgeries, or horizontally on primary surgeries, which matched an abdominal fold and at 2cm inferior to the location of the xiphoid appendix.

Dissection was performed in the necessary site on a supraperiostal plane, at the externum region and close to the ribs, coinciding to the skin limit marks. After dissection, the relief and dimensions of the internal space were assessed. That data would be used to sculpt the block posterior wall with scissors and scalpel, creating a mirror image of the osteocartilaginous structures relief. Afterwards, the block anterior face was sculpted, copying the sternum and ribs relief.

The last stage consisted of cutting the implant edges, aiming at smoothing and fitting the lateral edges to the space created. The success in fitting the block was measured by inserting the sculpted block and evaluating the external visual effect. Once the desired result was obtained, a 4.8-mm draining tube was positioned outside the abdominal midline, right below the incision. After the prosthesis was definitively inserted, two 2–0 nylon monofilament sutures were transfixed on the inferior edge of the block and fixed to the sternum. The subcutaneous tissue was sutured close with separate stitches using 4–0 absorbable thread, and the skin was sutured using continuous intradermal suture with 4–0 absorbable thread.

### Evaluation of results

The results were rated as satisfactory or unsatisfactory, according to the assessment noted on the patient's chart 120 days after the surgery. The cases that required revision surgery were rated again 120 days after the secondary surgery.

## RESULTS

This technique was used on 42 patients; 8 cases were excluded from this approach due to lack of data. The 34 remaining patients enrolled in this study were aged 15–54 years, mean age of 22 years and 3 months. For 26 patients it was their first surgery to repair *pectus excavatum*, while 8 of them had already undergone other types of procedures, as follows: 2 cases used the technique described by Ravitch,^(2)^ 2 used gelatinous silicone prosthesis, 2 underwent methacrylate injections with volumes of 100cc and 250cc according to the patient's report, and 2 had premolded and multiperforated hard solid silicone prosthesis. In 28 patients, incision was horizontal, 6–9-cm long. In two cases, the incision was over a prior medial scar: one incision was on the lateral margin of the chest over an oblique thoracic scar, and in another patient, a V-shaped incision on the sternal notch on the upper chest region, taking advantage of the existing scars from previous surgery incisions. The size of the blocks used varied from the smallest, measuring 7.5cm x 12cm on the base x 1.5-cm high, to the largest, 21cm x 19cm on the base x 3-cm high. Hardness index Shore A varied from 10 to 30, being 27 with 10 Shore A, 3 with 20 Shore A, and 2 with 30 Shore A.

Hematoma occurred on the second postoperative day on two patients, who were treated with methods using the existing drainage tube, not requiring new surgical intervention. Nine patients (26.4%) showed increased volume in the surgical site, 10 to 15 days after surgery, and percutaneous puncture was necessary to remove the serous and yellow fluid. The volume varied from 150 to 530mL; three to six punctures were needed during a maximum 10-day period. No case of infection was reported.

**Figure 1:**
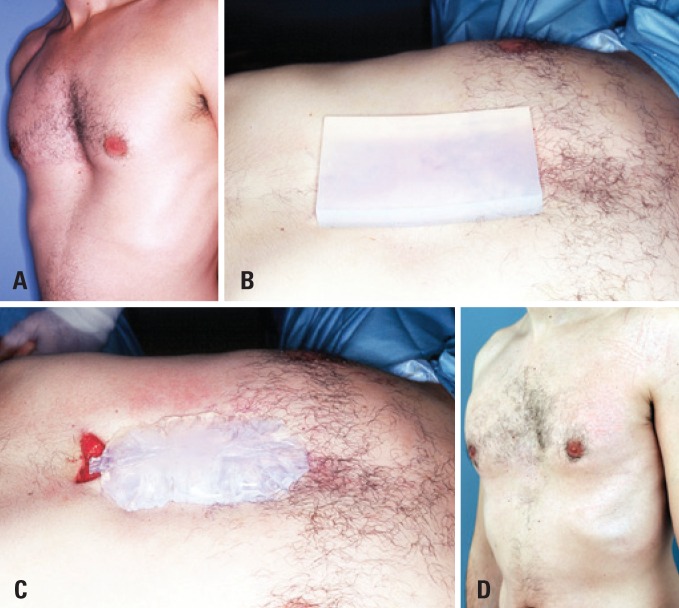
A 24 year-old patient with *pectus excavatum* presenting medial anterior chest depression underwent surgery on November 27, 2000. (A) Left oblique view. (B) Parallelepiped-shaped soft silicone block before sculpting. (C) Sculpted silicone block before final insertion. (D) Left oblique view 12 years after surgery

**Figure 2:**
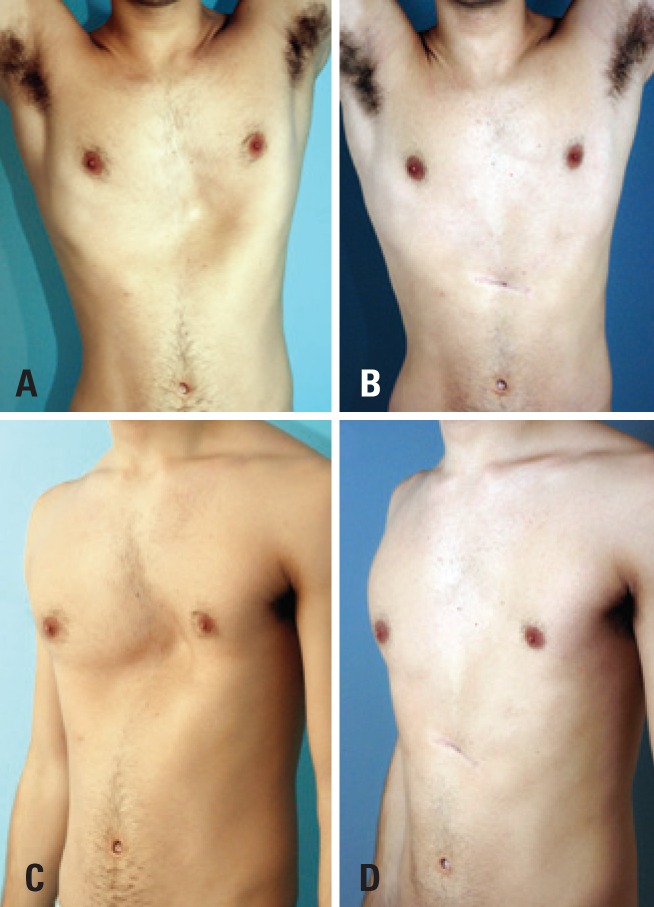
A 21 year-old patient with *pectus excavatum* presenting large depression on left ribs. (A) Frontal view. (B) Same patient 4 years after surgery. (C) Left oblique view. (D) Left lateral oblique view 4 years after surgery

Four patients underwent new surgical intervention. Two underwent autogenous fat graft to correct contour imperfections. One case presented relief defect on the prosthesis inferior portion and underwent surgery with local anesthesia for a better fit of the implant's dimensions. It consisted of removing a 5-mm x 10-mm fragment on the anterior and inferior margin of the prosthesis. In one case it was necessary to definitively remove the prosthesis because the patient complained of chronic pain on the ribs, which did not show inflammatory signs and it was not possible to make a diagnosis; pain relief was observed after removing silicone. The results were rated as satisfactory by 30 patients and unsatisfactory by 4. Of those four cases, three were re-rated as satisfactory 120 days after the secondary repair surgery was performed. Figures 1 and 2 show two cases with results rated as satisfactory.

## DISCUSSION

The first reports on correcting *pectus excavatum* with silicone (1970 and 1972) describe using an elastomer that was vulcanized at room temperature, 24 hours before the surgery, and prepared based on the mold from the existing bone defect.^(4–6)^ This type of silicone was banned in the late 1980's, because polymerization could cause unpredictable subcutaneous reactions after implant. Hence it was replaced by solid silicone prosthesis or premolded gelatinous-substance filled bags that were made based on the measures from the defect relief.^(7,8)^

These implants had two features that hinder the final aesthetic results: their reduced flexibility requiring extensive incisions to enable implanting them, and the impossibility of cutting the implant in order to adjust its fit. When inserting the implant, it was frequent to see that the measurements made during evaluation did not match the necessary actual dimensions.

In those cases that used gelatinous implants, the discrepant measures resulted in visible and palpable edges upon physical examination, which are even more visible during upper limb movements.^(9)^ The final external appearance shows rectified and relief-free skin because of the type of surface of the implants.

For this reason, we started using parallelepiped blocks that could be cut until achieving the size and contours similar to the internal area dissected during the surgery. Therefore, it is possible to copy the external contours and adjust the implant posterior surface to the irregularities on the osteocartilaginous contour, rendering the implant less prone to displacement.

**Figure 3:**
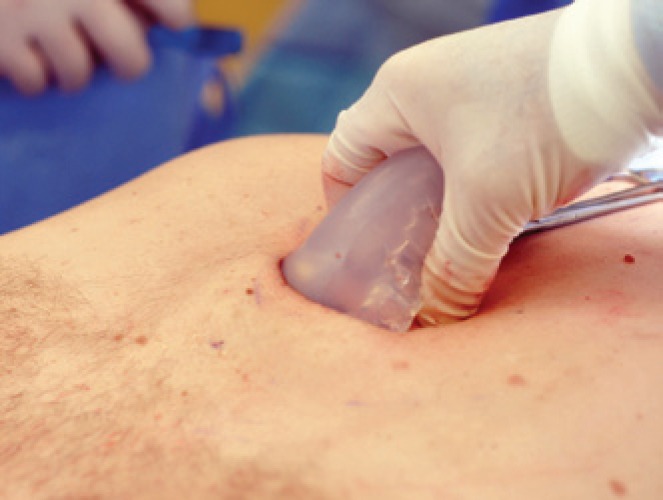
Illustration showing sculpted implant insertion made easier due to soft silicone block flexibility, which enables inserting through relatively small incisions

The manufacturing of solid silicone, hardness 10 Shore A started in 1994. This material is much more flexible than that used in other implants, which enabled smaller incisions and it was easier to insert (Figure 3). This allows inserting the block repeatedly in the dissected area to evaluate the intraoperative result. This type of material also allows trespassing suture needle and thread without the risk of tearing, thus fixating the prosthesis. Reports on the use of premolded solid silicone suggest making holes on the prosthesis to help fixating it for allowing repair tissue and fibrosis to penetrate,^(10)^ but that makes performing a secondary surgery more difficult, as observed in two cases of secondary surgery we performed.

The implant low hardness index helps the aesthetic result, hindering identification both visually or by touching over skin. The fact that it can be cut makes it possible to adjust a proper fit in height and the side margin relief, copying the ribs, which promotes a more natural-looking result.

The presence of a serous fluid in the late postoperative period was described when using silicone in patients that had *pectus excavatum* in 31% and 65% of the cases.^(9–11)^ It represented 26.7% of the cases we reviewed and did not compromise the results.

The patients who underwent fat grafts showed small irregularities on the edges of the prosthesis. In the only case in whom the result was totally lost due to implant removal because of chronic pain, it was not possible to make diagnosis, neither by exams nor by direct visual during removal surgery.

## CONCLUSION

The use of soft solid silicone block, ranging 10 to 30 Shore A, in surgical repair for *pectus excavatum* in male patients help achieve a better aesthetic result, since it is possible to sculpt the silicone block intraoperatively until reaching the desired dimensions and relief before the end of the procedure. This material flexibility enables using it with a small surgical incision that will suffer less trauma during the multiple insertions and removals of the implant during the procedure, as required to reach the final shape.
